# Prevalence and Characteristics of STRC Gene Mutations (DFNB16): A Systematic Review and Meta-Analysis

**DOI:** 10.3389/fgene.2021.707845

**Published:** 2021-09-21

**Authors:** Shuang Han, Dejun Zhang, Yingyuan Guo, Zeming Fu, Guofang Guan

**Affiliations:** Department of Otolaryngology Head and Neck Surgery, The Second Hospital of Jilin University, Changchun, China

**Keywords:** STRC gene, mutation, deafness, prevalence, meta-analysis

## Abstract

**Background:** Mutations in the STRC (MIM 606440) gene, inducing DFNB16, are considered a major cause of mild–moderate autosomal recessive non-syndromic hearing loss (ARNSHL). We conducted a systematic review and meta-analysis to determine the global prevalence and characteristics of STRC variations, important information required for genetic counseling.

**Methods:** PubMed, Google Scholar, Medline, Embase, and Web of Science were searched for relevant articles published before January 2021.

**Results:** The pooled prevalence of DFNB16 in GJB2-negative patients with hearing loss was 4.08% (95% CI: 0.0289–0.0573), and the proportion of STRC variants in the mild–moderate hearing loss group was 14.36%. Monoallelic mutations of STRC were 4.84% (95% CI: 0.0343–0.0680) in patients with deafness (non-GJB2) and 1.36% (95% CI: 0.0025–0.0696) in people with normal hearing. The DFNB16 prevalence in genetically confirmed patients (non-GJB2) was 11.10% (95% CI: 0.0716–0.1682). Overall pooled prevalence of deafness–infertility syndrome (DIS) was 36.75% (95% CI: 0.2122–0.5563) in DFNB16. The prevalence of biallelic deletions in STRC gene mutations was 70.85% (95% CI: 0.5824–0.8213).

**Conclusion:** Variants in the STRC gene significantly contribute to mild–moderate hearing impairment. Moreover, biallelic deletions are a main feature of STRC mutations. Copy number variations associated with infertility should be seriously considered when investigating DFNB16.

## Introduction

According to the World Health Organization, one in five people worldwide lives with hearing loss (HL)−5.5% of the population of the world (World Health Organization, 2021). Hearing problems can have a devastating impact on the mental health of the people and the ability to communicate, study, and even earn a living. Approximately half the cases of deafness have a genetic etiology (Sheffield and Smith, 2019). Although variations in gap junction protein beta 2 (GJB2) gene are the most common factor for prelingual, recessive deafness (50%), stereocilin (STRC) gene, known as DFNB16, is supposed to be another major contributor to bilateral mild-to-moderate hearing impairment (HI) (Francey et al., 2012; Yokota et al., 2019). Moreover, STRC mutations are considered the second most frequent cause associated with autosomal recessive non-syndromic hearing loss (ARNSHL) (Sloan-Heggen et al., 2016; Plevova et al., 2017; Back et al., 2019; Cada et al., 2019).

The STRC gene is located on chromosome 15q15.3 and named after its encoded protein—stereocilin—an extracellular structural protein expressed in the outer hair cells (OHCs) of the inner ear. Stereocilin was detected in six sensory areas in the inner ears of mice: the organ of Corti, the utricular maculae, the saccular maculae, and the three cristae ampullares of the vestibule (Verpy et al., 2001). Stereocilin was associated with OHCs in two cell-surface specializations interconnecting with the hair bundle in Corti contained with inner hair cells, OHCs, supporting cells, ciliated ends of the hair cells, and the tectorial membrane. The two specializations are the horizontal top connectors of adjacent stereocilia, which have a zipper-like structure, and the attachment links that anchor the tallest stereocilia into the overlying tectorial membrane (Verpy et al., 2008, 2011). In stereocilin null (Strc _/_) mice, both these links were absent, and progressive HL appeared from P15 (Verpy et al., 2008, 2011).

STRC has a tandem structure, and the linkage region includes three other genes: CATSPER2 (MIM 607249), PPIP5K1 (MIM 610979), and CKMT1B (MIM 123290) (Zhang et al., 2007). Causative alterations in the STRC gene include copy number variations (CNVs), single nucleotide variants (SNVs), or small insertions/deletions (indels). Recently, CNVs have been recognized as having an important role in STRC variations (Yokota et al., 2019). The STRC deletions are frequently accompanied by the deletion of the CATSPER2 gene accounting for sperm motility. This genotype, characterized by deletions including both CATSPER2 and STRC, is known as deafness–infertility syndrome (DIS) in both males and females (Hildebrand et al., 2009). STRC is part of a tandem duplication, and the second copy is a pseudogene (pSTRC). The highly homologous (>99%) distal pseudogene makes molecular analysis to detect STRC mutations by next-generation (NGS) and exome sequencing (ES) challenging (Vona et al., 2015; Shi et al., 2019). Reliable screening is especially significant and affordable in recent years due to the development of analytical approaches, such as MPLA, long-range/nested PCR, and droplet digital PCR (Back et al., 2019; Shi et al., 2019).

Many studies have routinely described genetic testing of patients with DFNB16. Compared with other deafness-associated gene variants, STRC has its own unique qualities and may lead to non-syndromic or syndromic deafness. However, to date, there is no publication systematically describing the prevalence and features of DFNB. Against this background, this meta-analysis provides a global and current pooled prevalence of STRC mutations based on available gene detection. Results generated from this paper will widen available options and contribute to genetic counseling for medical workers and individuals affected by HI.

## Methods

This search was performed following the guidelines of Preferred Reporting Items for Systematic Reviews and Meta-Analyses (PRISMA) (Moher et al., 2009) (Supplementary Table 1).

### Search Strategy

The literature search was conducted by electronic databases (PubMed, Google Scholar, Medline, Embase, and Web of Science) for the English language articles published prior to January 2021. We entered search terms (“STRC” OR “stereocilin” OR “DFNB16”) into each database. Two authors (SH and DZ) separately undertook literature searches and checked the reference lists of all selected articles. When disagreements occurred after the screening, further discussion took place to reach a consensus.

### Eligibility Criteria

We included researches that met the following criteria: (1) original research, (2) study population with HI and sample sizes with no <10 probands, (3) STRC gene detection, and (4) available full-text papers written in English. We excluded (1) duplicate publications, reviews, studies with overlapping data, mechanisms and/or animals, abstract-only articles, and texts without raw data; (2) fewer than 10 probands reported; and (3) studies published in languages other than English.

### Study Selection and Data Extraction

**Figure 1:**
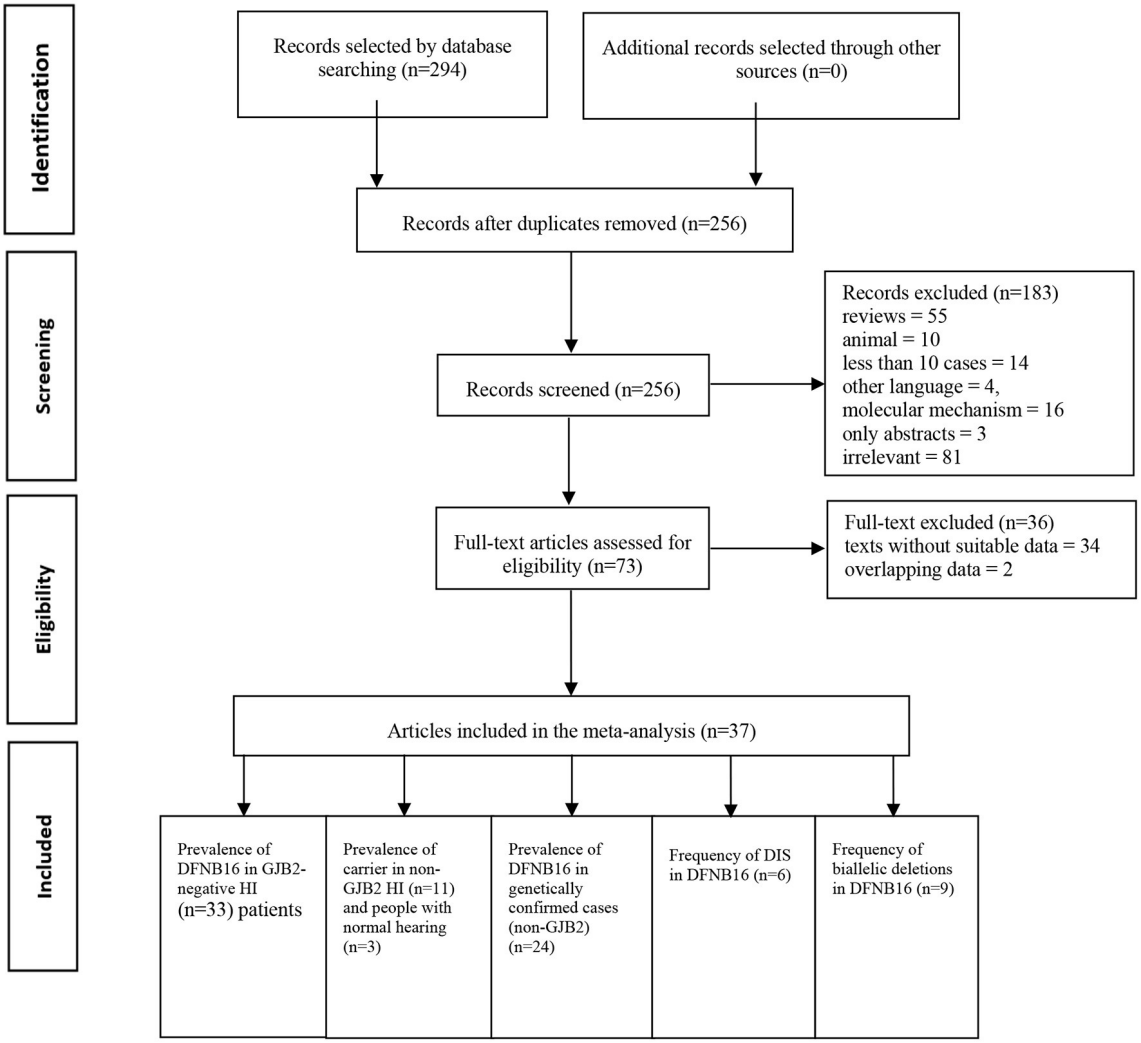
Standard data extraction diagram.

Two authors (SH and DZ) independently accomplished the literature selection based on predetermined criteria. The other researchers (YG and ZF) reviewed whether the results were consistent. If disagreements occurred, further discussion took place until a consensus was reached. A standard data extraction diagram is presented in Figure 1.

After relevant publications were selected, the data were collected by two reviewers (SH and DZ) from included papers as follows: first author, year of publication, region, study population, gene detection method, DFNB16, genetically confirmed cases (non-GJB2), total HI patients (non-GJB2), DIS, carriers in total HI patients (non-GJB2), carriers in the normal-hearing population, and types of mutations in STRC (biallelic CNVs, CNVs + SNVs, or small indels, biallelic SNVs, or small indels). Discrepancies were discussed and resolved by the senior author (GG).

### Quality Assessment

The risk of bias in each observational study was calculated using a tool developed by Hoy et al. (2012), with total scores ranging from 0 to 10. Bias was judged to be of low risk (9–10 points), moderate risk (6–8 points), or high risk (<6 points).

### Statistical Analysis

The meta-analysis was conducted using R (version 4.0.4, The R Foundation, Vienna, Austria). To bring the proportion data closer to a normal distribution, the logit transformation was used to solve the estimates < 0.2 or >0.8, while the double-arcsine method was chosen when extreme proportions (0 or 1) exist (Lipsey and Wilson, 2000). The Shapiro–Wilk normality test was applied to calculate the normal distribution of the transformed sample data. Assessments and 95% confidence intervals (CIs) of the prevalence of all collected articles were estimated by the random-effects model. Forest plots were used to show percentages of each study, the summary rate, and heterogeneity among publications. Between-study heterogeneity was evaluated by the *I*^2^ statistic. Meta-regression was calculated to investigate the potential source of high heterogeneity. The sensitivity analysis was completed by removing low-quality papers (score ≤ 5) and determining whether the results were stable. Funnel plots and Egger's bias test were used to assess the publication bias.

## Results

A total of 294 publications were extracted from the databases. After duplicates were excluded, titles and abstracts of the remaining articles were screened, and full-text versions of 73 relevant studies were further reviewed. Finally, 37 papers were included in the meta-analysis and were used in the subsets shown in Figure 1. All detailed information extracted from eligible articles is presented in Table 1, Supplementary Table 2. The prevalence of DFNB16 in HI patients (non-GJB2) was 4.08% (95% CI: 0.0289–0.0573), the prevalence of STRC carriers in the HI participants (non-GJB2) was 4.84% (95% CI: 0.0343–0.0680), and those with normal hearing accounted for 1.36% (95% CI: 0.0025–0.0696). The prevalence of DFNB16 in genetically confirmed cases (non-GJB2) was 11.10% (95% CI: 0.0716–0.1682), the prevalence of DIS in DFNB16 patients was 36.75% (95% CI: 0.2122–0.5563), and the prevalence of biallelic deletions in DFNB16 patients was 70.85% (95% CI: 0.5824–0.8213).

**Table 1:** Proportions extracted for meta-analysis.

**References**	**Region**	**Study population**	**Gene detection method^**#**^**	Frequency of DFNB16^*^	**Carrier-frequency in non-GJB2 HI**	**Carrier-frequency in people with normal hearing**	Frequency of DFNB16 in confirmed cases^**^	**Frequency of DIS in DFNB16**	**Types of mutations in STRC**		
									**CNVs**	**CNVs+ NVs or indels**	**SNVs or indels**
Sheppard et al. (2018)	USA	HL	NGS+CNV	2.50%	2.50%		7.14%				
Lebeko et al. (2016)	Cameroon	ARNSHL	NGS+CNV	10.00%							
Marková et al. (2018)	Czech	NSHL	NGS+CNV	5.56%	4.51%			37.50%	50.00%	43.75	6.25
Plevova et al. (2017)	Czech	HL	NGS+CNV	10.20%			35.71%				
Chang and Choi (2014)	Korea	HL	NGS	0.88%			1.56%				
Safka Brozkova et al. (2020)	Czech	NSHL	NGS+CNV	5.23%	2.61%		40.74%		50.00%	36.36%	13.64%
Kim et al. (2020)	Korea	HL	NGS+CNV	36.71%			60.42%	44.83%	58.62%	34.48%	6.90%
Schrauwen et al. (2013)	Europe	ARNSHL	NGS	8.33%							
Kannan-Sundhari et al. (2020)	Iran	HL	NGS	4.35%							
Ito et al. (2019)	Japan	NSHL	NGS+CNV	5.95%	2.38%	0.93%					
Back et al. (2019)	Germany	ARNSHL	NGS+CNV	10.98%							
Mehta et al. (2016)	USA	NSHL	NGS+CNV	2.41%			37.14%				
Morgan et al. (2020)	Italy	NSHL	NGS+CNV	7.53%				21.21%			
García-García et al. (2020)	Spain	HL	NGS+CNV	2.75%	0.92%		7.89%				
Morgan et al. (2018)	Italy	NSHL	NGS+CNV	1.94%			6.25%				
Francey et al. (2012)	USA	NSHL	NGS+CNV	2.58%		0.52%			52.94%	29.41%	17.65%
Gu et al. (2015)	China	NSHL	NGS+CNV	1.59%							
Yokota et al. (2019)	Japan	NSHL	NGS+CNV	1.95%	5.28%	2.63%	7.05%	88.24%	100%		
Downie et al. (2020)	Australia	HL	NGS+CNV	4.76%			10.81%				
Sommen et al. (2016)	Western-European	ARNSHL	NGS+CNV	0.76%	8.40%		3.45%				
Zazo Seco et al. (2017)	Netherlands	HL	NGS+CNV	2.09%			6.90%				
Vona et al. (2015)	Germany	NSHL	NGS+CNV	6.38%	5.32%						
Shearer et al. (2014)	USA	HL	NGS+CNV					10.81%	83.78%	16.22%	
Budde et al. (2020)	Egypt	NSHL	NGS+CNV	1.75%			2.27%				
Cabanillas et al. (2018)	Spain	HL	NGS+CNV	4.00%	8.00%		9.52%				
Moteki et al. (2016)	Japan	NSHL	NGS+CNV	1.55%			5.77%				
Sloan-Heggen et al. (2016)	USA	HL	NGS+CNV	6.93%			20.58%	23.94%	77.46%	21.13%	1.41%
Mandelker et al. (2014)	NA	HL	NGS+CNV						63.64%	36.36%	
Bademci et al. (2016)	Multiple	ARNSHL	NGS+CNV	0.63%			1.11%				
Baux et al. (2017)	France	NSHL	NGS+CNV	5.70%			18.00%				
Ji et al. (2014)	China	HL	NGS+CNV		11.27%						
Costales et al. (2020)	Spain	HL	NGS	4.55%			10.00%				
Sloan-Heggen et al. (2015)	Iran	HL	NGS+CNV	0.33%			0.50%				
Guan et al. (2018)	USA	NSHL	NGS+CNV	8.00%			18.18%				
Amr et al. (2018)	NA	HL	NGS+CNV					35.48%	74.19%	22.58%	3.23%
Shearer et al. (2013)	USA	NSHL	NGS+CNV	4.26%	1.06%		11.11%				
Brownstein et al. (2020)	Israel	HL	NGS	2.27%			3.77%				

# *NGS including: panel, targeted testing, ES, exome sequencing; CES, clinical exome sequencing; WES, whole exome sequencing; TGE, targeted genome enrichment; Sanger also included; CNV including: MLPA, QF-PCR, long-range/nested PCR, microdroplet PCR, droplet digital PCR, array CGH, SNP microarray, CMA, chromosomal microarray analysis; QCF PCR, quantitative comparative fluorescent PCR; MPS, massively parallel sequencing.*

* *The frequency of STRC is achieved from GJB2-negative patients.*

** *Genetically confirmed cases except for GJB2-related.*

### Quality Assessment

The details of quality assessment scores for each article are available in Supplementary Table 3. None of the included studies received low risk for items 2, 3, and 9, as the cluster sampling method, random selection, census, or the length of the shortest prevalence period were not provided in each survey. Out of the remaining 7 possible points, 4 studies received 7 points, 13 studies obtained 6 points, and 20 studies received 5 points.

### The Global Prevalence of DFNB16 in GJB2-Negative Hearing Impaired Patients

**Figure 2:**
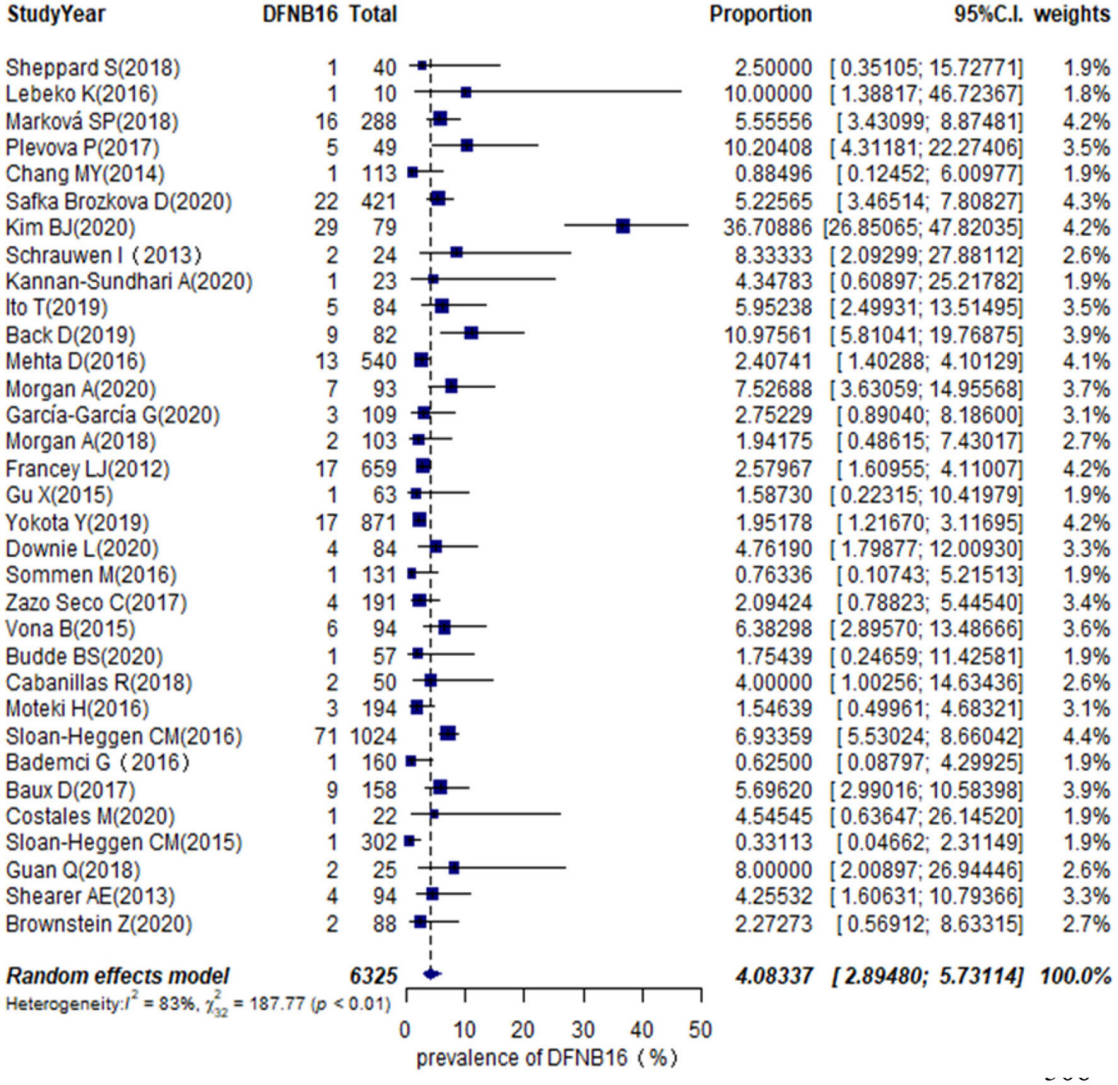
The global prevalence of DFNB16 in GJB2-negative hearing impaired (HI) patients.

The proportion of STRC mutations in GJB2-irrelevant HI patients varied from 0.33 to 36.71% among 33 studies (Table 1), with the highest rate observed in Korea (Kim et al., 2020). The overall pooled prevalence was 4.08% (95% CI: 0.0289–0.0573, *I*^2^ = 83%, *p* < 0.01) using a random-effects model among 6,325 subjects (Figure 2). The *I*^2^ and *p*-value indicated substantial heterogeneity.

Meta-regression was used to estimate the source of heterogeneity. We investigated five categorical moderators: region (Europe, Asia, America, or others), study population (HL, NSHL, or ARNSHL), gene detection method (NGS or NGS+CNV), degree of HI (mild–moderate or other HI), and quality assessment grade (score ≤ 5 or score >5). Significant estimates were not found for moderators in region, study population, gene detection method, and quality assessment (*p* = 0.9351, 0.8068, 0.6876, 0.8419). The degree of HI was significantly associated with the overall pooled prevalence (*p* = 0.0003). The *R*^2^ (amount of heterogeneity accounted for) was 49.91%, meaning that the degree of HI can explain about 49.91% of heterogeneity in the DFNB16 prevalence among GJB2-negative HI patients.

The prevalence of STRC mutations in GJB2-negative HI patients was further analyzed by subgroup focusing on world region (Supplementary Figure 1A) and the degree of HI (Supplementary Figure 1B). For region, the highest rate estimated was in Europe, which was 5.40% (95% CI: 0.0409–0.0711), followed by the US at 3.94% (95% CI: 0.0222–0.0690), other regions with 3.00% (95% CI: 0.0103–0.0841), and Asia at 2.77% (95% CI: 0.0075–0.0965). The estimated prevalence of mild–moderate HI was 14.36% (95% CI: 0.0365–0.4259), and that of other HI was 3.67% (95% CI: 0.0281–0.0478).

**Figure 3:**
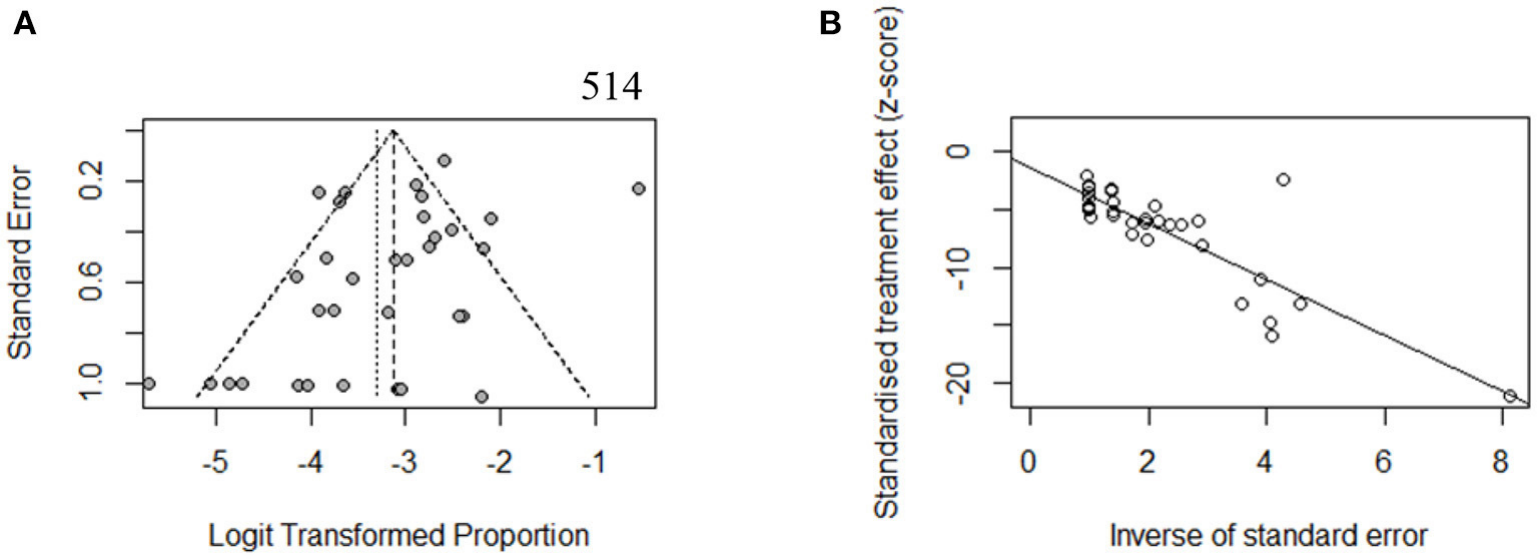
**(A)** Funnel plot. **(B)** Egger test.

To estimate the stability of outcomes, we conducted sensitivity analyses by assessing the effects of removing low-quality publications (Francey et al., 2012; Chang and Choi, 2014; Gu et al., 2015; Vona et al., 2015; Bademci et al., 2016; Mehta et al., 2016; Sommen et al., 2016; Baux et al., 2017; Zazo Seco et al., 2017; Cabanillas et al., 2018; Guan et al., 2018; Marková et al., 2018; Sheppard et al., 2018; Back et al., 2019; Budde et al., 2020; Downie et al., 2020; Kim et al., 2020; Safka Brozkova et al., 2020). The summary prevalence of DFNB16 in non-GJB2 HL patients was 3.98% (95% CI: 0.0260–0.0606, *I*^2^ = 70%, *p* < 0.01), which stabilized the findings in the range of that of crude analysis. The *I*^2^ and *p*-value also indicated substantial heterogeneity. Results from a funnel plot (Figure 3A) and Egger test (*p* = 0.0549, Figure 3B) indicate an insignificant level of publication bias.

### Prevalence of Carrier STRC Mutation in Non-GJB2 Hearing Impaired Participants and Individuals With Normal Hearing

**Figure 4:**
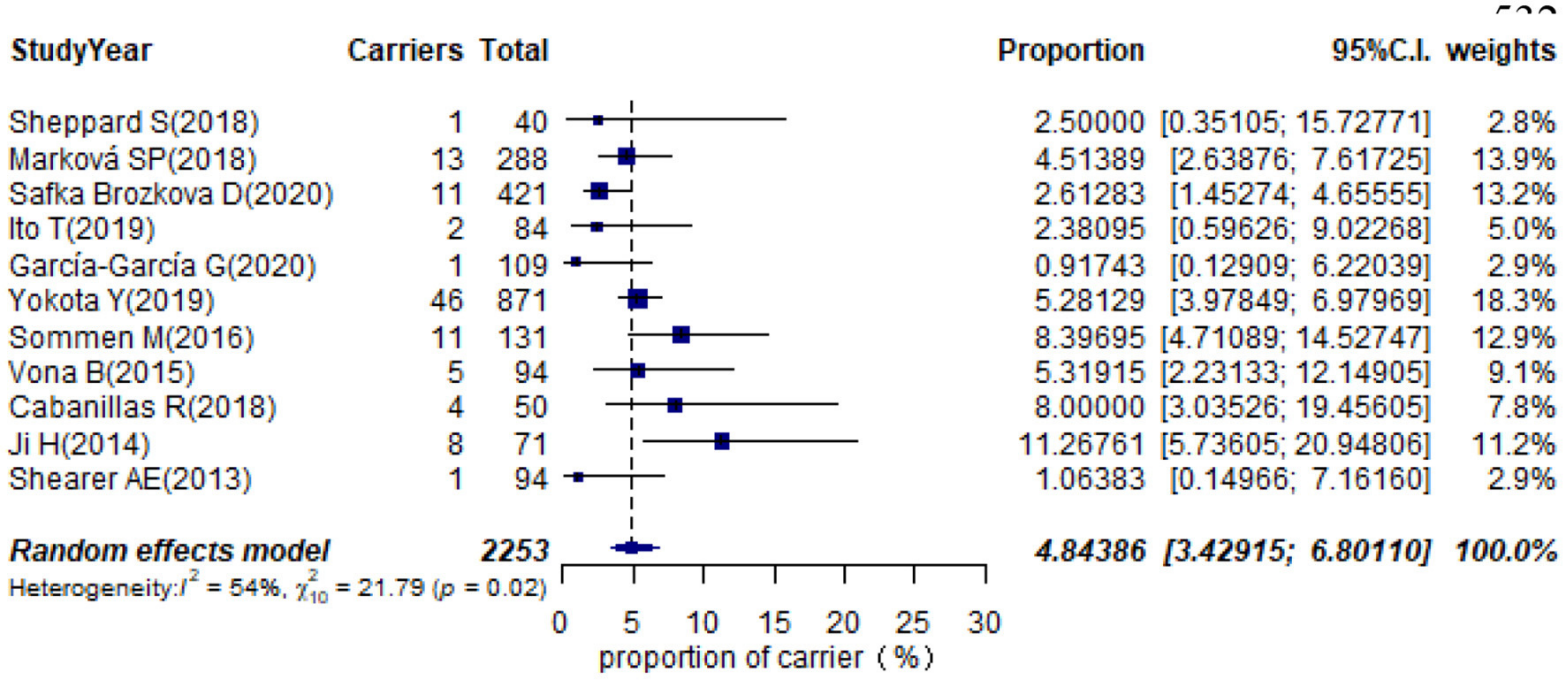
Prevalence of carrier STRC mutation in non-GJB2 HI participants.

**Figure 5:**
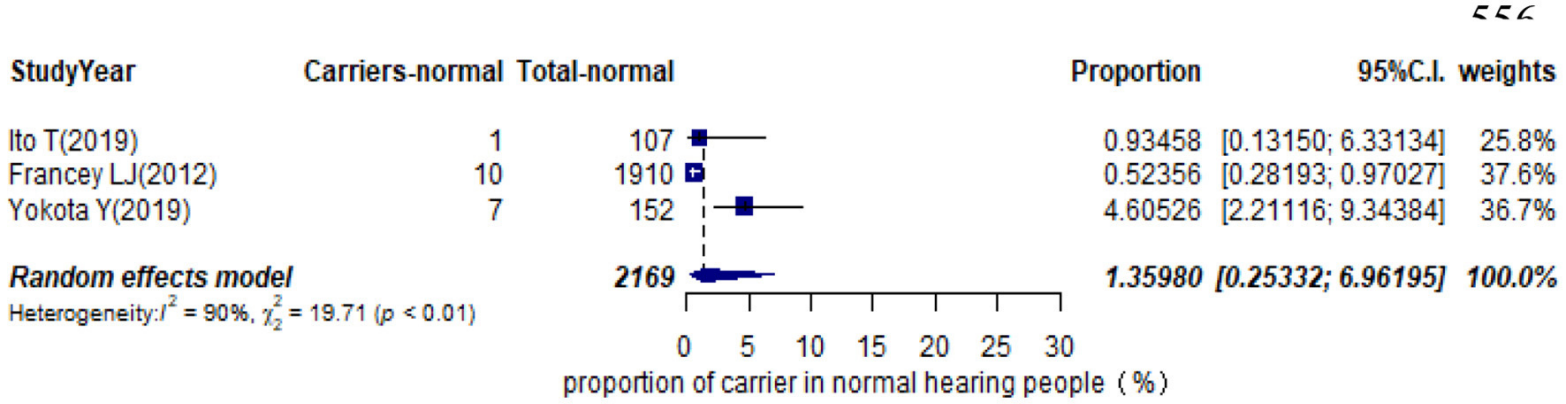
Prevalence of carrier STRC mutation in normal hearing people.

Eleven studies presented monoallelic variants of the STRC gene in non-GJB2 HI participants, with carriers varying from 0.92% in Spain to 11.27% in China (Table 1). The pooled carrier prevalence in HI participants (non-GJB2) assessed by the random-effects model was 4.84% (95% CI: 0.0343–0.0680, *I*^2^ = 54%, Figure 4). Three studies showed the carrier of the STRC mutation among normal-hearing individuals (Table 1). The summary carrier prevalence in people with normal hearing was 1.36% (95% CI: 0.0025–0.0696, *I*^2^ = 90%, Figure 5) using a random-effects model.

### Prevalence of DFNB16 Among Genetically Confirmed Cases (Non-GJB2)

**Figure 6:**
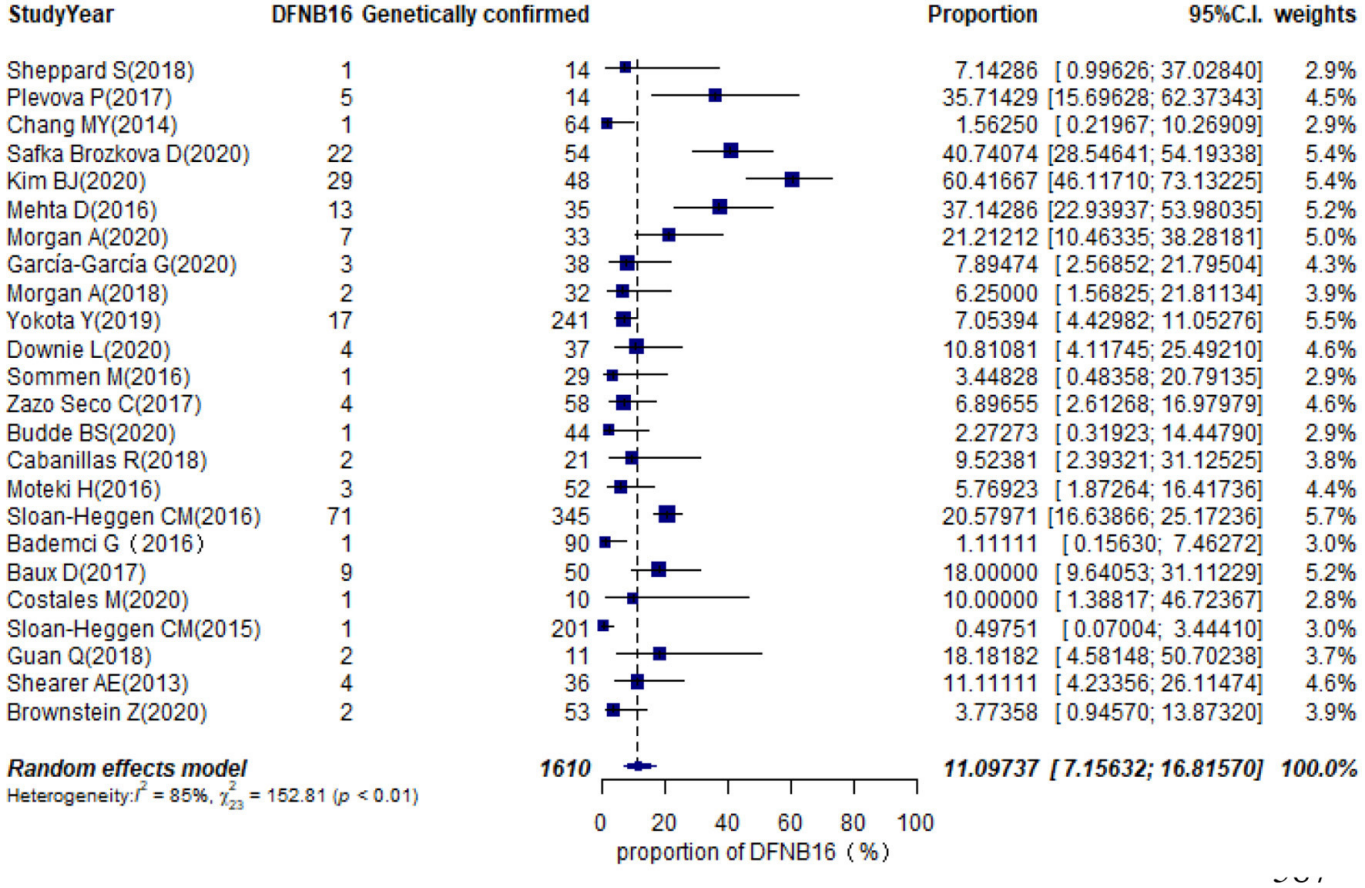
Prevalence of DFNB16 among genetically confirmed cases (non-GJB2).

Twenty-eight studies described genetically confirmed patients (Supplementary Table 2). Twenty-four were included in the meta-analysis to estimate the prevalence of STRC mutations in genetically diagnosed patients (non-GJB2) because the sample sizes of those with genetic diagnoses in these publications were no <10 cases (Table 1). The prevalence achieved using the random-effects model was 11.10% (95% CI: 0.0716–0.1682, *I*^2^ = 85%, *p* < 0.01) in 1,610 GJB2-negative genetically confirmed cases (Figure 6). *I*^2^ and *p*-value indicated substantial heterogeneity. Meta-regression was applied to evaluate the source of heterogeneity, with five categorical moderators: region, study population, gene detection method, degree of HI, and grade of quality assessment. Significant values were not detected for region, study population, gene detection method, and quality assessment (*p* = 0.9596, 0.5827, 0.1221, 0.4449). The degree of HI accounted for 28.00% of the variance between studies (*p* = 0.0019, *R*^2^ = 28.00%). The subgroup by region is shown in Supplementary Figure 2A, where the highest was in the US at 20.48% (95% CI: 0.1277–0.3118), followed by Europe at 14.54% (95% CI: 0.0820–0.2448), Asia with 5.68% (95% CI: 0.0113–0.2402), and other regions at 3.69% (95% CI: 0.0080–0.1535). The estimated pooled prevalence in the mild–moderate HI group was 50.71% (95% CI: 0.2813–0.7300) and that of the other-HI group was 9.44% (95% CI: 0.0620–0.1412) (Supplementary Figure 2B). Sensitivity analyses were completed by assessing the effects of deleting low-quality studies (Chang and Choi, 2014; Bademci et al., 2016; Mehta et al., 2016; Sommen et al., 2016; Baux et al., 2017; Zazo Seco et al., 2017; Cabanillas et al., 2018; Guan et al., 2018; Sheppard et al., 2018; Budde et al., 2020; Downie et al., 2020; Kim et al., 2020; Safka Brozkova et al., 2020). The summary prevalence of DFNB16 in genetically confirmed (non-GJB2) HL patients was 9.63% (95% CI: 0.0558–0.1614, *I*^2^ = 80%, *p* < 0.01) in the sensitivity analysis and without apparent fluctuation. The *I*^2^ and *p*-value showed substantial heterogeneity.

### Prevalence of Deafness–Infertility Syndrome in STRC-Associated Hearing Impairment

**Figure 7:**
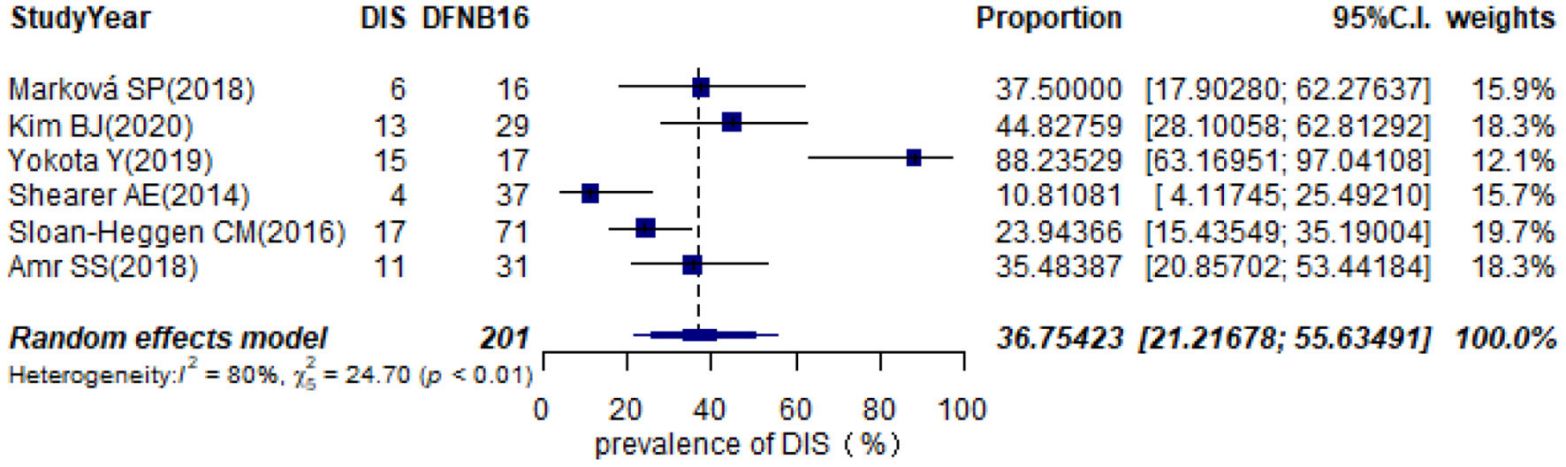
Prevalence of deafness–infertility syndrome (DIS) in DFNB16.

Twelve studies provided information about homozygous deletion in the CATSPER2 gene (Supplementary Table 2). Six articles, including ≥10 DFNB16 patients, were chosen for meta-analysis (Table 1). The overall pooled DIS prevalence in DFNB16 was 36.75% (95% CI: 0.2122–0.5563, *I*^2^ = 80%) using a random-effects model among 201 individuals (Figure 7).

### Prevalence of Biallelic Deletions in DFNB16

**Figure 8:**
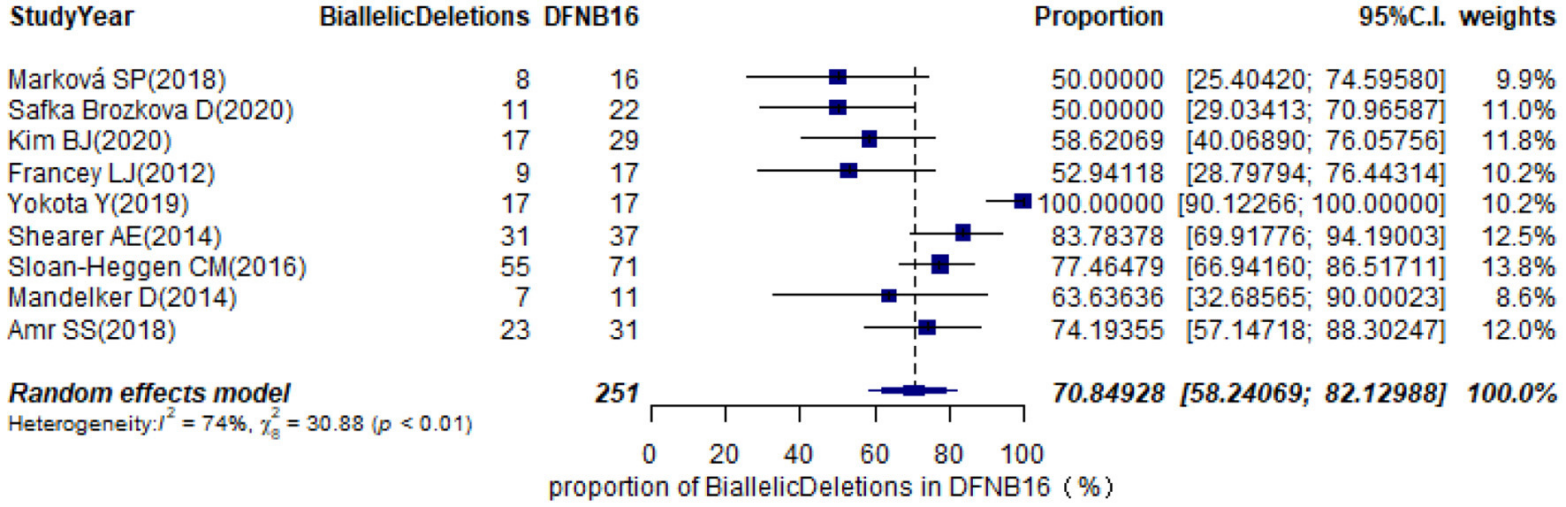
Prevalence of biallelic deletions in DFNB16.

The types of mutations in STRC were described in 36 studies (Supplementary Table 2). Of these, we selected nine articles with sample sizes not smaller than 10 DFNB16 cases for meta-analysis (Table 1). Because data extracted from Yokota et al. (2019) had proportions equal to 1, we analyzed the raw data with double arcsine transformation in advance. The pooled prevalence of biallelic deletions in DFNB16 was 70.85% (95% CI: 0.5824–0.8213, *I*^2^ = 74%) with a random-effects model (Figure 8).

## Discussion

The classical two-step strategy for deafness genetic testing consists of GJB2/6 locus analysis and gene panel based on NGS technologies. Although most panels have the STRC gene to detect SNVs or small indels, it is challenging for the NGS dataset to accurately detect CNVs in STRC, and even more challenging to detect CATSPER2 (Yokota et al., 2019). Considering the high prevalence of DFNB16 in genetically confirmed cases (11.10%), especially in the mild–moderate HI subgroup (50.71%), and biallelic CNVs in DFNB16 (70.85%), we consider that CNV detection of STRC should be accompanied by panel testing in case of misdiagnosis. Furthermore, males with DFNB16 should be advised about CATSPER2 gene sequencing because the DIS prevalence in DFNB16 was 36.75%. This is essential information that should be considered during genetic counseling.

Different multistep strategies have been implemented for genetic exploration of HL. Valuable data were generated and screened in our study, and information should be extracted according to the following three aspects to make outcomes more reliable: First, as far as possible, consanguineous individuals should be merged into families. For instance, Lebeko et al. (2016) included 26 individuals from 10 GJB2-negative families, and the DFNB16 proportion was based on 10 families rather than patients. Although Mehta et al. (Mehta et al., 2016) did not describe consanguinity precisely, we did not omit the study from the analysis because it offered a large sample with the number of families marginally less than individuals. Second, apart from deletions and gene-pseudogene conversions, we also identified heterozygous duplications as monoallelic mutations, although there was no evidence of whether the duplications were pathogenic or had any effect on phenotypes (Yokota et al., 2019). At present, no patient has been diagnosed with biallelic duplications. Variations in pseudogenes that mutated into an inactive form and SNVs classified as non-pathogenic or benign were excluded for pooling prevalence. Third, when we estimated the prevalence of DFNB16 in genetically confirmed cases (non-GJB2), DIS in DFNB16, and biallelic deletions in STRC-associated HI patients, we removed studies with probands <10, which accords with the sample size exclusion criteria mentioned. From databases and references, only Cada et al. (2019) and Markova et al. (2020) showed more than 10 DFNB16 sample sizes for clinical features of DFNB16. However, we rejected these studies because it was unclear whether the data presented were for all the original patients or only the cases selected after qualified audiological examination was completed. There were no other eligible articles to add.

When separating by HI degree, the prevalence of DFNB16 in non-GJB2 patients was significantly higher in the mild–moderate group (14.36%) than the other-HI group (3.67%) whose degree of HI was unclear or mixed. In the same way, the prevalence of DFNB16 in genetically confirmed cases (non-GJB2) was significantly higher in the mild–moderate group (50.71%) compared with the other-HI group (9.40%). Our results emphasized that STRC is a primary contributor to mild–moderate HI. This conclusion has also been documented in previous research (Francey et al., 2012; Yokota et al., 2019; Kim et al., 2020).

Our data show that diallelic CNVs (mainly deletions) are an extreme factor in STRC gene mutations and are probably accompanied by homozygous deletions in CATSPER2 gene (36.75%) simultaneously. Given that STRC CNVs might be ignored in studies using NGS HL panels, screening techniques that contain CNV detection of STRC and CATSPER2 are recommended for patients before NGS analyses, especially in patients with bilateral mild-to-moderate HI (Plevova et al., 2017). Males with DIS will be deaf and infertile, and this is crucial information that should be realized during genetic counseling (Yokota et al., 2019). Females who inherit homozygous STRC-CATSPER2 deletions will only be deaf (Hildebrand et al., 2009), but CATSPER2 CNVs in women should also be taken seriously, not only to identify the etiology in probands but also to predict and prevent the disability in the next generation.

There are some limitations to this meta-analysis. First, studies of the prevalence of STRC-related patients were available from only 16 countries, and data were not equally distributed. There were insufficient publications to provide adequate information for some continents. For example, in Africa, data were derived exclusively from Cameroon and Egypt. There were only five studies from developing countries, likely due to limited medical recording systems and medical care linked to possible under-recognition and late diagnosis of this disease. Second, the study designs and population covered in the included studies varied from hospital-based studies to national research. Investigation of DFNB16 in different populations and settings is urgently needed. Moreover, sample sizes of research studies need to be expanded, and more funding will be required for large-scale studies. Third, there is no uniform detection method for STRC variations. Information about STRC CNVs, monoallelic mutation, or CATSPERS2 deletions was ignored in some studies, affecting our confidence in prevalence assessment. The screening of CNVs could be impacted by different CNV detection methods, such as NGS or SNP array, and the proportion of STRC mutations may be found to be higher if effective strategies are applied (Yokota et al., 2019).

In conclusion, we undertook the first meta-analysis to demonstrate that DFNB16 plays a crucial role in mild-to-moderate ARNSHL. The findings also emphasize the significance of detecting copy number variations of the STRC gene.

## Data Availability Statement

The original contributions presented in the study are included in the article/Supplementary Material, further inquiries can be directed to the corresponding author.

## Author Contributions

SH conducted literature searches and data extraction and wrote the manuscript. DZ conducted literature searches and data extraction. YG and ZF reviewed the results. GG determined the final outcome, and wrote and checked the manuscript. All authors contributed to the article and approved the submitted version.

## Conflict of Interest

The authors declare that the research was conducted in the absence of any commercial or financial relationships that could be construed as a potential conflict of interest.

## Publisher's Note

All claims expressed in this article are solely those of the authors and do not necessarily represent those of their affiliated organizations, or those of the publisher, the editors and the reviewers. Any product that may be evaluated in this article, or claim that may be made by its manufacturer, is not guaranteed or endorsed by the publisher.

## References

[B1] Amr S. S. Murphy E. Duffy E. Niazi R. Balciuniene J. Luo M.. (2018). Allele-specific droplet digital PCR combined with a next-generation sequencing-based algorithm for diagnostic copy number analysis in genes with high homology: proof of concept using Stereocilin. Clin. Chem. 64, 705–714. 10.1373/clinchem.2017.280685 29339441

[B2] Back D. Shehata-Dieler W. Vona B. Hofrichter M. A. H. Schroeder J. Haaf T.. (2019). Phenotypic characterization of DFNB16-associated hearing loss. Otol. Neurotol. 40, e48–e55. 10.1097/MAO.0000000000002059 30531641

[B3] Bademci G. Foster J. Mahdieh N. Bonyadi M. Duman D. Cengiz F. B.. (2016). Comprehensive analysis via exome sequencing uncovers genetic etiology in autosomal recessive nonsyndromic deafness in a large multiethnic cohort. Genet. Med. 18, 364–371. 10.1038/gim.2015.89 26226137 PMC4733433

[B4] Baux D. Vaché C. Blanchet C. Willems M. Baudoin C. Moclyn M.. (2017). Combined genetic approaches yield a 48% diagnostic rate in a large cohort of French hearing-impaired patients. Sci. Rep. 7:16783. 10.1038/s41598-017-16846-9 29196752 PMC5711943

[B5] Brownstein Z. Gulsuner S. Walsh T. Martins F. T. A. Taiber S. Isakov O.. (2020). Spectrum of genes for inherited hearing loss in the Israeli Jewish population, including the novel human deafness gene ATOH1. Clin. Genet. 98, 353–364. 10.1111/cge.13817 33111345 PMC8045518

[B6] Budde B. S. Aly M. A. Mohamed M. R. Bre,ß A Altmüller J. Motameny S.. (2020). Comprehensive molecular analysis of 61 Egyptian families with hereditary non-syndromic hearing loss. Clin. Genet. 98, 32–42. 10.1111/cge.13754 32279305

[B7] Cabanillas R. Diñeiro M. Cifuentes G. A. Castillo D. Pruneda P. C. Álvarez R.. (2018). Comprehensive genomic diagnosis of non-syndromic and syndromic hereditary hearing loss in Spanish patients. BMC Med. Genomics. 11:58. 10.1186/s12920-018-0375-5 29986705 PMC6038346

[B8] Cada Z Šafka BroŽková D. Balatková Z. Plevová P. Rašková D. Laštuvková J.. (2019). Moderate sensorineural hearing loss is typical for DFNB16 caused by various types of mutations affecting the STRC gene. Eur. Arch. Otorhinolaryngol. 276, 3353–3358. 10.1007/s00405-019-05649-5 31552524

[B9] Chang M. Y. Choi B. Y. (2014). Strategy for the customized mass screening of genetic sensorineural hearing loss in Koreans. Korean J Audiol. 18, 45–49. 10.7874/kja.2014.18.2.45 25279224 PMC4181059

[B10] Costales M. Diñeiro M. Cifuentes G. A. Capín R. Otero A. Viejo-Díaz M.. (2020). Clinical utility of next-generation sequencing in the aetiological diagnosis of sensorineural hearing loss in a Childhood Hearing Loss Unit. Acta Otorrinolaringol. Esp. 71, 166–174. 10.1016/j.otoeng.2019.05.005 31706454

[B11] Downie L. Halliday J. Burt R. Lunke S. Lynch E. Martyn M.. (2020). Exome sequencing in infants with congenital hearing impairment: a population-based cohort study. Eur. J. Hum. Genet. 28, 587–596. 10.1038/s41431-019-0553-8 31827275 PMC7171096

[B12] Francey L. J. Conlin L. K. Kadesch H. E. Clark D. Berrodin D. Sun Y.. (2012). Genome-wide SNP genotyping identifies the Stereocilin (STRC) gene as a major contributor to pediatric bilateral sensorineural hearing impairment. Am. J. Med. Genet. A 158A, 298–308. 10.1002/ajmg.a.34391 22147502 PMC3264741

[B13] García-García G. Berzal-Serrano A. García-Díaz P. Villanova-Aparisi R. Juárez-Rodríguez S. de Paula-Vernetta C.. (2020). Improving the management of patients with hearing loss by the implementation of an NGS panel in clinical practice. Genes (Basel) 11:1467. 10.3390/genes11121467 33297549 PMC7762334

[B14] Gu X. Guo L. Ji H. Sun S. Chai R. Wang L.. (2015). Genetic testing for sporadic hearing loss using targeted massively parallel sequencing identifies 10 novel mutations. Clin. Genet. 87, 588–593. 10.1111/cge.12431 24853665

[B15] Guan Q. Balciuniene J. Cao K. Fan Z. Biswas S. Wilkens A.. (2018). AUDIOME: a tiered exome sequencing-based comprehensive gene panel for the diagnosis of heterogeneous nonsyndromic sensorineural hearing loss. Genet. Med. 20, 1600–1608. 10.1038/gim.2018.48 29595809

[B16] Hildebrand M. S. Avenarius M. R. Smith R. J. H. (2009). *CATSPER*-related male infertility, in GeneReviews^®^, eds Adam M. P. Ardinger HH H. H. Pagon R. A. Wallace S. E. Bean L. J. H Mirzaa G. Amemiya A. (Seattle, WA: University of Washington), 1993–2021. 20301780

[B17] Hoy D. Brooks P. Woolf A. Blyth F. March L. Bain C.. (2012). Assessing risk of bias in prevalence studies: modification of an existing tool and evidence of interrater agreement. J. Clin. Epidemiol. 65, 934–939. 10.1016/j.jclinepi.2011.11.014 22742910

[B18] Ito T. Kawashima Y. Fujikawa T. Honda K. Makabe A. Kitamura K.. (2019). Rapid screening of copy number variations in STRC by droplet digital PCR in patients with mild-to-moderate hearing loss. Hum. Genome Var. 30:41. 10.1038/s41439-019-0075-5 31645979 PMC6804619

[B19] Ji H. Lu J. Wang J. Li H. Lin X. (2014). Combined examination of sequence and copy number variations in human deafness genes improves diagnosis for cases of genetic deafness. BMC Ear Nose Throat Disord. 14:9. 10.1186/1472-6815-14-9 25342930 PMC4194081

[B20] Kannan-Sundhari A. Yan D. Saeidi K. Sahebalzamani A. Blanton S. H. Liu X. Z. (2020). Screening consanguineous families for hearing loss using the MiamiOtoGenes panel. Genet. Test. Mol. Biomarkers 24, 674–680. 10.1089/gtmb.2020.0153 32991204 PMC7585618

[B21] Kim B. J. Oh D. Y. Han J. H. Oh J. Kim M. Y. Park H. R.. (2020). Significant Mendelian genetic contribution to pediatric mild-to-moderate hearing loss and its comprehensive diagnostic approach. Genet. Med. 22, 1119–1128. 10.1038/s41436-020-0774-9 32203226

[B22] Lebeko K. Sloan-Heggen C. M. Noubiap J. J. Dandara C. Kolbe D. L. Ephraim S. S.. (2016). Targeted genomic enrichment and massively parallel sequencing identifies novel nonsyndromic hearing impairment pathogenic variants in Cameroonian families. Clin. Genet. 90, 288–290. 10.1111/cge.12799 27246798 PMC5324826

[B23] Lipsey M. Wilson D. B. (2000). Practical Meta-Analysis. London: SAGE Publications.

[B24] Mandelker D. Amr S. S. Pugh T. Gowrisankar S. Shakhbatyan R. Duffy E.. (2014). Comprehensive diagnostic testing for stereocilin: an approach for analyzing medically important genes with high homology. J. Mol. Diagn. 16, 639–647. 10.1016/j.jmoldx.2014.06.003 25157971

[B25] Marková S. P. BroŽková D. Š. Laššuthová P. Mészárosová A. Krutová M. Neupauerová J.. (2018). STRC gene mutations, mainly large deletions, are a very important cause of early-onset hereditary hearing loss in the Czech population. Genet. Test. Mol. Biomarkers 22, 127–134. 10.1089/gtmb.2017.0155 29425068

[B26] Markova T. G. Alekseeva N. N. Mironovich O. L. Galeeva N. M. Lalayants M. R. Bliznetz E. A.. (2020). Clinical features of hearing loss caused by STRC gene deletions/mutations in Russian population. Int. J. Pediatr. Otorhinolaryngol. 138:110247. 10.1016/j.ijporl.2020.110247 32705992

[B27] Mehta D. Noon S. E. Schwartz E. Wilkens A. Bedoukian E. C. Scarano I.. (2016). Outcomes of evaluation and testing of 660 individuals with hearing loss in a pediatric genetics of hearing loss clinic. Am. J. Med. Genet. A 170, 2523–2530. 10.1002/ajmg.a.37855 27480936

[B28] Moher D. Liberati A. Tetzlaff J. Altman D. J. PRISMA Group (2009). Preferred reporting items for systematic reviews and meta-analyses: the PRISMA statement. PLoS Med. 21:e1000097. 10.1371/journal.pmed.1000097 19621072 PMC2707599

[B29] Morgan A. Lenarduzzi S. Cappellani S. Pecile V. Morgutti M. Orzan E.. (2018). Genomic studies in a large cohort of hearing impaired Italian patients revealed several new alleles, a rare case of uniparental disomy (UPD) and the importance to search for copy number variations. Front. Genet. 9:681. 10.3389/fgene.2018.00681 30622556 PMC6309105

[B30] Morgan A. Lenarduzzi S. Spedicati B. Cattaruzzi E. Murru F. M. Pelliccione G.. (2020). Lights and shadows in the genetics of syndromic and non-syndromic hearing loss in the Italian population. Genes (Basel) 22:1237. 10.3390/genes11111237 33105617 PMC7690429

[B31] Moteki H. Azaiez H. Booth K. T. Shearer A. E. Sloan C. M. Kolbe D. L.. (2016). Comprehensive genetic testing with ethnic-specific filtering by allele frequency in a Japanese hearing-loss population. Clin. Genet. 89, 466–472. 10.1111/cge.12677 26346818 PMC4783301

[B32] Plevova P. Paprskarova M. Tvrda P. Turska P. Slavkovsky R. Mrazkova E. (2017). STRC deletion is a frequent cause of slight to moderate congenital hearing impairment in the Czech Republic. Otol. Neurotol. 38, e393–e400. 10.1097/MAO.0000000000001571 28984810

[B33] Safka Brozkova D. Poisson Marková S. Mészárosová A. U. Jenčík J. Cejnová V. Cada Z.. (2020). Spectrum and frequencies of non GJB2 gene mutations in Czech patients with early non-syndromic hearing loss detected by gene panel NGS and whole-exome sequencing. Clin. Genet. 98, 548–554. 10.1111/cge.13839 32860223

[B34] Schrauwen I. Sommen M. Corneveaux J. J. Reiman R. A. Hackett N. J. Claes C.. (2013). A sensitive and specific diagnostic test for hearing loss using a microdroplet PCR-based approach and next generation sequencing. Am. J. Med. Genet. A 161A, 145–152. 10.1002/ajmg.a.35737 23208854

[B35] Shearer A. E. Black-Ziegelbein E. A. Hildebrand M. S. Eppsteiner R. W. Ravi H. Joshi S.. (2013). Advancing genetic testing for deafness with genomic technology. J. Med. Genet. 50, 627–634. 10.1136/jmedgenet-2013-101749 23804846 PMC3887546

[B36] Shearer A. E. Kolbe D. L. Azaiez H. Sloan C. M. Frees K. L.. (2014). Copy number variants are a common cause of non-syndromic hearing loss. Genome Med. 6:37. 10.1186/gm554 24963352 PMC4067994

[B37] Sheffield A. M. Smith R. J. H. (2019). The epidemiology of deafness. Cold Spring Harb. Perspect. Med. 9:a033258. 10.1101/cshperspect.a033258 30249598 PMC6719589

[B38] Sheppard S. Biswas S. Li M. H. Jayaraman V. Slack I. Romasko E. J.. (2018). Utility and limitations of exome sequencing as a genetic diagnostic tool for children with hearing loss. Genet. Med. 20, 1663–1676. 10.1038/s41436-018-0004-x 29907799 PMC6295269

[B39] Shi L. Bai Y. Kharbutli Y. Oza A. M. Amr S. S. Edelmann L.. (2019). Prenatal cytogenomic identification and molecular refinement of compound heterozygous STRC deletion breakpoints. Mol. Genet. Genomic Med. 7:e806. 10.1002/mgg3.806 31218851 PMC6687617

[B40] Sloan-Heggen C. M. Babanejad M. Beheshtian M. Simpson A. C. Booth K. T. Ardalani F.. (2015). Characterising the spectrum of autosomal recessive hereditary hearing loss in Iran. J. Med. Genet. 52, 823–829. 10.1136/jmedgenet-2015-103389 26445815 PMC4733363

[B41] Sloan-Heggen C. M. Bierer A. O. Shearer A. E. Kolbe D. L. Nishimura C. J. Frees K. L.. (2016). Comprehensive genetic testing in the clinical evaluation of 1119 patients with hearing loss. Hum. Genet. 135, 441–450. 10.1007/s00439-016-1648-8 26969326 PMC4796320

[B42] Sommen M. Schrauwen I. Vandeweyer G. Boeckx N. Corneveaux J. J. van den Ende J.. (2016). DNA diagnostics of hereditary hearing loss: a targeted resequencing approach combined with a mutation classification system. Hum. Mutat. 37, 812–819. 10.1002/humu.22999 27068579

[B43] Verpy E. Leibovici M. Michalski N. Goodyear R. J. Houdon C. Weil D.. (2011). Stereocilin connects outer hair cell stereocilia to one another and to the tectorial membrane. J. Comp. Neurol. 519, 194–210. 10.1002/cne.22509 21165971 PMC3375590

[B44] Verpy E. Masmoudi S. Zwaenepoel I. Leibovici M. Hutchin T. P. Del Castillo I.. (2001). Mutations in a new gene encoding a protein of the hair bundle cause nonsyndromic deafness at the DFNB16 locus. Nat. Genet. 29, 345–349. 10.1038/ng726 11687802

[B45] Verpy E. Weil D. Leibovici M. Goodyear R. J. Hamard G. Houdon C.. (2008). Stereocilin-deficient mice reveal the origin of cochlear waveform distortions. Nature 456, 255–258. 10.1038/nature07380 18849963 PMC3338146

[B46] Vona B. Hofrichter M. A. Neuner C. Schröder J. Gehrig A. Hennermann J. B.. (2015). DFNB16 is a frequent cause of congenital hearing impairment: implementation of STRC mutation analysis in routine diagnostics. Clin. Genet. 87, 49–55. 10.1111/cge.12332 26011646 PMC4302246

[B47] World Health Organization (2021). Deafness and Hearing Loss. Available online at: https://www.who.int/health-topics/hearing-loss#tab=tab_1 (accessed April 15, 2021).

[B48] Yokota Y. Moteki H. Nishio S. Y. Yamaguchi T. Wakui K. Kobayashi Y.. (2019). Frequency and clinical features of hearing loss caused by STRC deletions. Sci. Rep. 9:4408. 10.1038/s41598-019-40586-7 30867468 PMC6416315

[B49] Zazo Seco C. Wesdorp M. Feenstra I. Pfundt R. Hehir-Kwa J. Y. Lelieveld S. H.. (2017). The diagnostic yield of whole-exome sequencing targeting a gene panel for hearing impairment in The Netherlands. Eur. J. Hum. Genet. 25, 308–314. 10.1038/ejhg.2016.182 28000701 PMC5315517

[B50] Zhang Y. Malekpour M. Al-Madani N. Kahrizi K. Zanganeh M. Lohr N. J.. (2007). Sensorineural deafness and male infertility: a contiguous gene deletion syndrome. J. Med. Genet. 44, 233–240. 10.1136/jmg.2006.045765 17098888 PMC2598039

