# Supplementary material for: Prevalence and Characteristics of STRC Gene Mutations (DFNB16): A Systematic Review and Meta-Analysis
Source: Front Genet. 2021 Sep 21;12:707845. doi: 10.3389/fgene.2021.707845 (PMC8491653; doi:10.3389/fgene.2021.707845)
Supplement: Supplementary file 1 [file Data_Sheet_1.docx]

Supplementary Material

Supplementary Figure 1a.

The STRC mutations prevalence in GJB2-negative HI patients analyzed by subgroup focused on world region


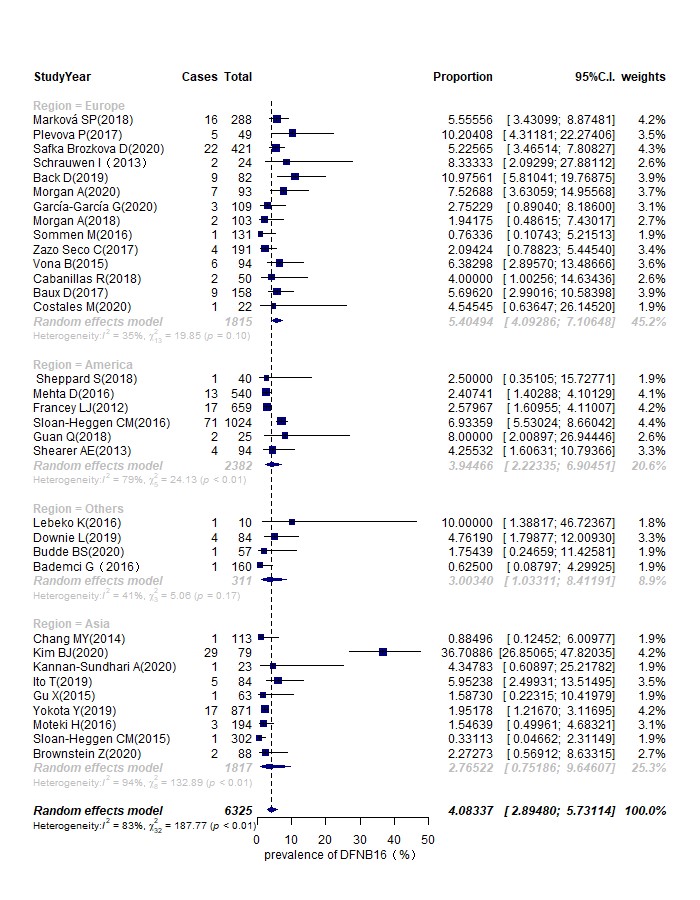


Supplementary Figure 1b.

The STRC mutations prevalence in GJB2-negative HI patients analyzed by subgroup focused on the grade of HI


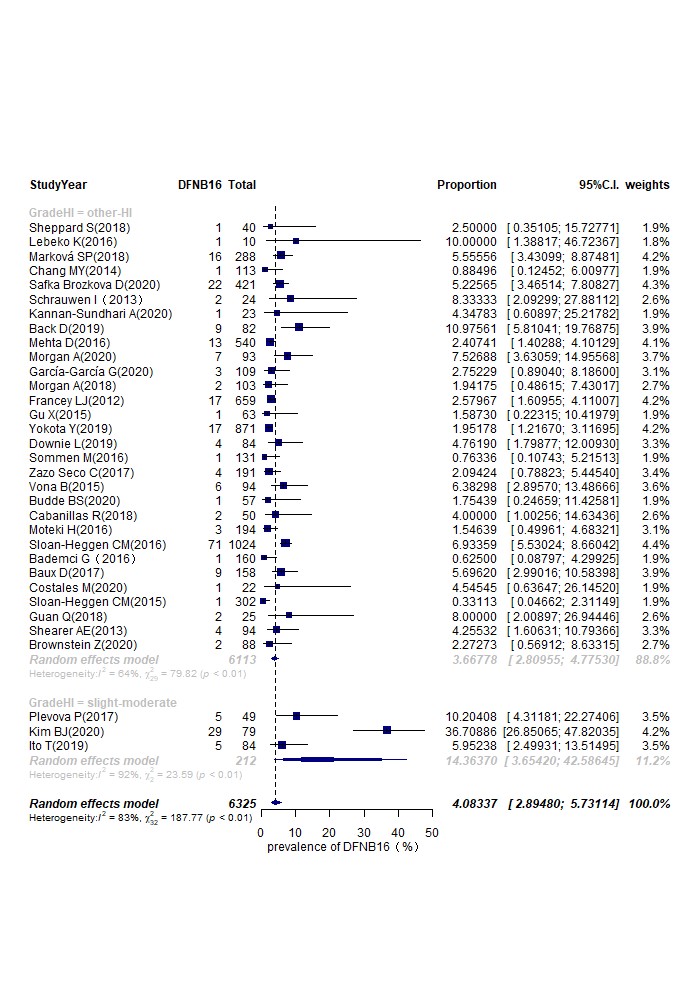


Supplementary Figure 2a.


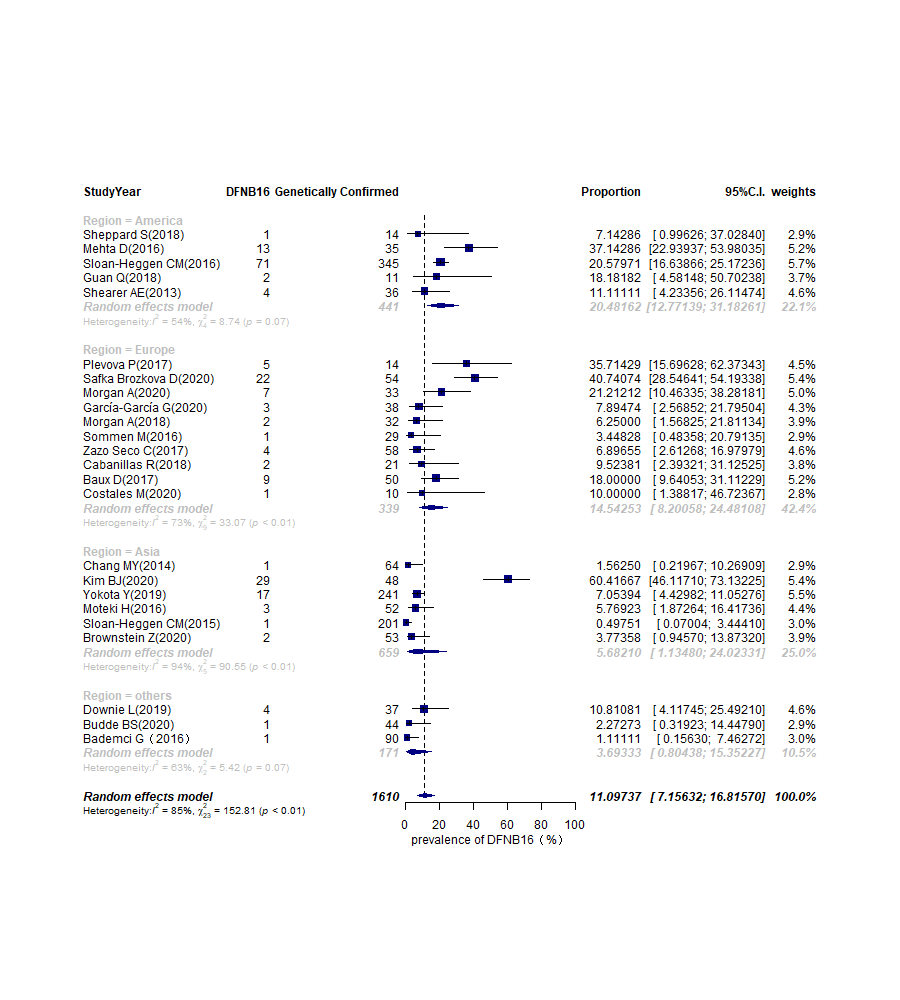
The DFNB16 prevalence in genetically confirmed cases (non-GJB2) analyzed by subgroup focused on world region

Supplementary Figure 2b


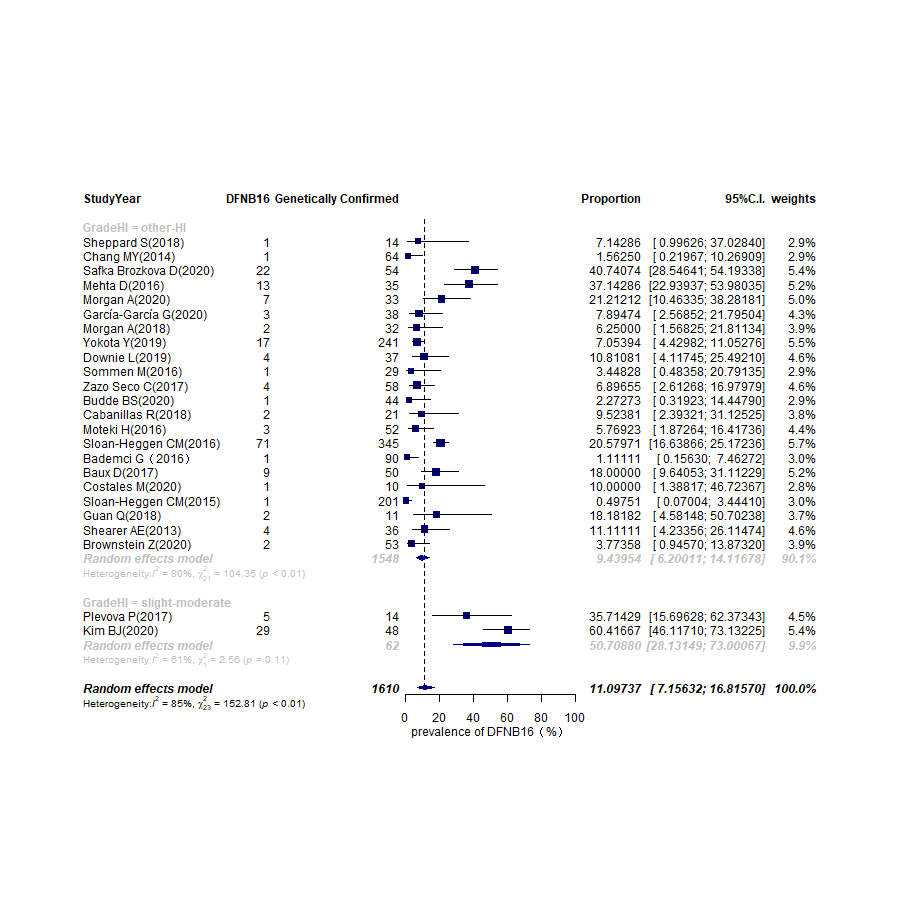
The DFNB16 prevalence in genetically confirmed cases (non-GJB2) analyzed by subgroup focused on the grade of HI
