# Supplementary material for: Prevalence and Characteristics of STRC Gene Mutations (DFNB16): A Systematic Review and Meta-Analysis
Source: Front Genet. 2021 Sep 21;12:707845. doi: 10.3389/fgene.2021.707845 (PMC8491653; doi:10.3389/fgene.2021.707845)
Supplement: Supplementary file 3 [file Table_2.doc]

Supplementary Table 2 Data collected from each study

| First Author | Year | Region | Study population | Gene detection method | DFNB16 | Genetically confirmed cases * | Total HI patients* | DIS | Carriers # | Carriers in normal hearing population | Types of mutations in STRC | | |
| --- | --- | --- | --- | --- | --- | --- | --- | --- | --- | --- | --- | --- | --- |
|  |  |  |  |  |  |  |  |  |  |  | CNVs | CNVs + SNVs or indel | SNVs or indel |
| Sheppard S | 2018 | USA | HL | NGS+CNV | 1 | 14 | 40 |  | 1 |  | 0 | 0 | 1 |
| Lebeko K | 2016 | Cameroon | ARNSHL | NGS+CNV | 1 | 7 | 10 |  |  |  | 1 | 0 | 0 |
| Marková SP | 2018 | Czech | NSHL | NGS+CNV | 16 |  | 288 | 6 | 13 |  | 8 | 7 | 1 |
| Plevova P | 2017 | Czech | HL | NGS+CNV | 5 | 14 | 49 |  |  |  | 4 | 1 | 0 |
| Chang MY | 2014 | Korea | HL | NGS | 1 | 64 | 113 |  |  |  | 0 | 0 | 1 |
| Safka Brozkova D | 2020 | Czech | NSHL | NGS+CNV | 22 | 54 | 421 |  | 11 |  | 11 | 8 | 3 |
| Kim BJ | 2020 | Korea | HL | NGS+CNV | 29 | 48 | 79 | 13 |  |  | 17 | 10 | 2 |
| Schrauwen I | 2013 | Europe | ARNSHL | NGS | 2 | 9 | 24 |  |  |  | 0 | 0 | 2 |
| Kannan-Sundhari A | 2020 | Iran | HL | NGS | 1 | 9 | 23 |  |  |  | 0 | 0 | 1 |
| Ito T | 2019 | Japan | NSHL | NGS+CNV | 5 |  | 84 |  | 2 | 1/107 | 2 | 3 | 0 |
| Back D | 2019 | Germany | ARNSHL | NGS+CNV | 9 |  | 82 | 1 |  |  | 3 | 6 | 1 |
| Mehta D | 2016 | USA | NSHL | NGS+CNV | 13 | 35 | 540 |  |  |  |  |  |  |
| Morgan A | 2020 | Italy | NSHL | NGS+CNV | 7 | 33 | 93 |  |  |  | 6 | 0 | 1 |
| García-García G | 2020 | Spain | HL | NGS+CNV | 3 | 38 | 109 | 3 | 1 |  | 3 |  |  |
| Morgan A | 2018 | Italy | NSHL | NGS+CNV | 2 | 32 | 103 | 1 |  |  | 2 |  |  |
| Francey LJ | 2012 | USA | NSHL | NGS+CNV | 17 |  | 659 |  |  | 10/1910 | 9 | 5 | 3 |
| Gu X | 2015 | China | NSHL | NGS+CNV | 1 | 8 | 63 | 1 |  |  | 1 |  |  |
| Yokota Y | 2019 | Japan | NSHL | NGS+CNV | 17 | 241 | 871 | 15 | 46 | 7/152 | 17 |  |  |
| Downie L | 2020 | Australia | HL | NGS+CNV | 4 | 37 | 84 |  |  |  | 3 |  | 1 |
| Sommen M | 2016 | Western-  European | ARNSHL | NGS+CNV | 1 | 29 | 131 |  | 11 |  |  | 1 |  |
| Zazo Seco C | 2017 | Netherlands | HL | NGS+CNV | 4 | 58 | 191 |  |  |  | 4 |  |  |
| Vona B | 2015 | Germany | NSHL | NGS+CNV | 6 |  | 94 |  | 5 |  | 2 | 3 | 1 |
| Shearer AE | 2014 | USA | HL | NGS+CNV | 37 |  |  | 4 |  |  | 31 | 6 |  |
| Budde BS | 2020 | Egypt | NSHL | NGS+CNV | 1 | 44 | 57 |  |  |  |  |  | 1 |
| Cabanillas R | 2018 | Spain | HL | NGS+CNV | 2 | 21 | 50 |  | 4 |  | 2 |  |  |
| Moteki H | 2016 | Japan | NSHL | NGS+CNV | 3 | 52 | 194 | 2 |  |  | 3 |  |  |
| Sloan-Heggen CM | 2016 | USA | HL | NGS+CNV | 71 | 345 | 1024 | 17 |  |  | 55 | 15 | 1 |
| Mandelker D | 2014 | NA | HL | NGS+CNV | 11 |  |  |  |  |  | 7 | 4 |  |
| Bademci G | 2016 | Multilpy | ARNSHL | NGS+CNV | 1 | 90 | 160 |  |  |  | 1 |  |  |
| Baux D | 2017 | France | NSHL | NGS+CNV | 9 | 50 | 158 |  |  |  | 4 | 3 | 2 |
| Ji H | 2014 | China | HL | NGS+CNV |  |  | 71 |  | 8 |  |  |  |  |
| Costales M | 2020 | Spain | HL | NGS | 1 | 10 | 22 | 1 |  |  | 1 |  |  |
| Sloan-Heggen CM | 2015 | Iran | HL | NGS+CNV | 1 | 201 | 302 |  |  |  | 1 |  |  |
| Guan Q | 2018 | USA | NSHL | NGS+CNV | 2 | 11 | 25 |  |  |  | 2 |  |  |
| Amr SS | 2018 | NA | HL | NGS+CNV | 31 |  |  | 11 |  |  | 23 | 7 | 1 |
| Shearer AE | 2013 | USA | NSHL | NGS+CNV | 4 | 36 | 94 |  | 1 |  | 3 | 1 |  |
| Brownstein Z | 2020 | Israel | HL | NGS | 2 | 53 | 88 |  |  |  | 1 | 1 |  |

* except for GJB2 HI patients

# Carriers in HI patients(non-GJB2)
