# Supplementary material for: Prevalence and Characteristics of STRC Gene Mutations (DFNB16): A Systematic Review and Meta-Analysis
Source: Front Genet. 2021 Sep 21;12:707845. doi: 10.3389/fgene.2021.707845 (PMC8491653; doi:10.3389/fgene.2021.707845)
Supplement: Supplementary file 4 [file Table_3.doc]

Supplementary Table 3 Risk of bias in each study

| Risk of Bias Tool | | | | | | | | | | | | | |
| --- | --- | --- | --- | --- | --- | --- | --- | --- | --- | --- | --- | --- | --- |
|  |  |  | External Validity | | | | Internal Validity | | | | | | score |
|  |  |  | 1 | 2 | 3 | 4 | 5 | 6 | 7 | 8 | 9 | 10 |  |
| 1 | Sheppard S | Sheppard S(2018) | N | NA | NA | Y | Y | Y | Y | N | NA | Y | 5 |
| 2 | Lebeko K | Lebeko K(2016) | N | NA | NA | Y | Y | Y | Y | Y | NA | Y | 6 |
| 3 | Marková SP | Marková SP(2018) | N | NA | NA | Y | Y | Y | Y | N | NA | Y | 5 |
| 4 | Plevova P | Plevova P(2017) | N | NA | NA | Y | Y | Y | Y | Y | NA | Y | 6 |
| 5 | Chang MY | Chang MY(2014) | N | NA | NA | Y | Y | Y | Y | N | NA | Y | 5 |
| 6 | Safka Brozkova D | Safka Brozkova D(2020) | N | NA | NA | Y | Y | Y | Y | N | NA | Y | 5 |
| 7 | Kim BJ | Kim BJ(2020) | N | NA | NA | Y | Y | Y | Y | N | NA | Y | 5 |
| 8 | Schrauwen I | Schrauwen I（2013） | N | NA | NA | Y | Y | Y | Y | Y | NA | Y | 6 |
| 9 | Kannan-Sundhari A | Kannan-Sundhari A(2020) | N | NA | NA | Y | Y | Y | Y | Y | NA | Y | 6 |
| 10 | Ito T | Ito T(2019) | N | NA | NA | Y | Y | Y | Y | Y | NA | Y | 6 |
| 11 | Back D | Back D(2019) | N | NA | NA | Y | Y | Y | Y | N | NA | Y | 5 |
| 12 | Mehta D | Mehta D(2016) | N | NA | NA | Y | Y | Y | Y | N | NA | Y | 5 |
| 13 | Morgan A | Morgan A(2020) | Y | NA | NA | Y | Y | Y | Y | N | NA | Y | 6 |
| 14 | García-García G | García-García G(2020) | N | NA | NA | Y | Y | Y | Y | Y | NA | Y | 6 |
| 15 | Morgan A | Morgan A(2018) | Y | NA | NA | Y | Y | Y | Y | Y | NA | Y | 7 |
| 16 | Francey LJ | Francey LJ(2012) | N | NA | NA | Y | Y | Y | Y | N | NA | Y | 5 |
| 17 | Gu X | Gu X(2015) | N | NA | NA | Y | Y | Y | Y | N | NA | Y | 5 |
| 18 | Yokota Y | Yokota Y(2019) | Y | NA | NA | Y | Y | Y | Y | Y | NA | Y | 7 |
| 19 | Downie L | Downie L(2020) | N | NA | NA | Y | Y | Y | Y | N | NA | Y | 5 |
| 20 | Sommen M | Sommen M(2016) | N | NA | NA | Y | Y | Y | Y | N | NA | Y | 5 |
| 21 | Zazo Seco C | Zazo Seco C(2017) | N | NA | NA | Y | Y | Y | Y | N | NA | Y | 5 |
| 22 | Vona B | Vona B(2015) | N | NA | NA | Y | Y | Y | Y | N | NA | Y | 5 |
| 23 | Shearer AE | Shearer AE(2014) | N | NA | NA | Y | Y | Y | Y | N | NA | Y | 5 |
| 24 | Budde BS | Budde BS(2020) | N | NA | NA | Y | Y | Y | Y | N | NA | Y | 5 |
| 25 | Cabanillas R | Cabanillas R(2018) | N | NA | NA | Y | Y | Y | Y | N | NA | Y | 5 |
| 26 | Moteki H | Moteki H(2016) | Y | NA | NA | Y | Y | Y | Y | Y | NA | Y | 7 |
| 27 | Sloan-Heggen CM | Sloan-Heggen CM(2016) | N | NA | NA | Y | Y | Y | Y | Y | NA | Y | 6 |
| 28 | Mandelker D | Mandelker D(2014) | N | NA | NA | Y | Y | Y | Y | Y | NA | Y | 6 |
| 29 | Bademci G | Bademci G（2016） | N | NA | NA | Y | Y | Y | Y | N | NA | Y | 5 |
| 30 | Baux D | Baux D(2017) | N | NA | NA | Y | Y | Y | Y | N | NA | Y | 5 |
| 31 | Ji H | Ji H(2014) | N | NA | NA | Y | Y | Y | Y | N | NA | Y | 5 |
| 32 | Costales M | Costales M(2020) | N | NA | NA | Y | Y | Y | Y | Y | NA | Y | 6 |
| 33 | Sloan-Heggen CM | Sloan-Heggen CM(2015) | N | NA | NA | Y | Y | Y | Y | Y | NA | Y | 6 |
| 34 | Guan Q | Guan Q(2018) | N | NA | NA | Y | Y | Y | Y | N | NA | Y | 5 |
| 35 | Amr SS | Amr SS(2018) | N | NA | NA | Y | Y | Y | Y | Y | NA | Y | 6 |
| 36 | Shearer AE | Shearer AE(2013) | N | NA | NA | Y | Y | Y | Y | Y | NA | Y | 6 |
| 37 | Brownstein Z | Brownstein Z(2020) | Y | NA | NA | Y | Y | Y | Y | Y | NA | Y | 7 |
